# Psychopathology in Substance Use Disorder Patients with and without Substance-Induced Psychosis

**DOI:** 10.1155/2015/843762

**Published:** 2015-08-31

**Authors:** Simon Zhornitsky, Andràs Tikàsz, Élie Rizkallah, Jean-Pierre Chiasson, Stéphane Potvin

**Affiliations:** ^1^Department of Clinical Neurosciences and Hotchkiss Brain Institute, Faculty of Medicine, University of Calgary, 2500 University Dr. NW, Calgary, AB, Canada T2N 1N4; ^2^Centre de recherche de l'Institut Universitaire en Santé Mentale de Montréal, 7331 Hochelage Street, Montreal, QC, Canada H3P 3E5; ^3^Department of Psychiatry, University of Montreal, 2900 Boulevard Édouard-Montpetit, Montreal, QC, Canada H3T 1J4; ^4^Department of Psychology, Université du Québec à Montréal, 100 Sherbrooke West Street, Montreal, QC, Canada H2X 3P2; ^5^Clinique du Nouveau Départ, 1110 Avenue Beaumont, Montreal, QC, Canada H3P 3E5

## Abstract

*Background*. Substance-induced psychotic disorder (SIPD) is a diagnosis constructed to distinguish substance-induced psychotic states from primary psychotic disorders. A number of studies have compared SIPD persons with primary psychotic patients, but there is little data on what differentiates substance use disorder (SUD) individuals with and without SIPD. Here, we compared psychopathology, sociodemographic variables, and substance use characteristics between SUD patients with and without SIPD. *Methods*. A retrospective chart review was conducted on newly admitted patients at a rehabilitation centre between 2007 and 2012. *Results*. Of the 379 patients included in the study, 5% were diagnosed with SIPD (*n* = 19) and 95% were diagnosed with SUDs without SIPD (*n* = 360). More SIPD patients reported using cannabis and psychostimulants, and fewer SIPD patients reported using alcohol than SUDs patients without SIPD. SIPD patients scored higher on the “schizophrenia nuclear symptoms” dimension of the SCL-90R psychoticism scale and exhibited more ClusterB personality traits than SUD patients without SIPD. *Discussion*. These data are consistent with previous studies suggesting that psychopathology, substance type, and sociodemographic variables play important role in the development of SIPD. More importantly, the results highlight the need for paying greater attention to the types of self-reported psychotic symptoms during the assessment of psychotomimetic effects associated with psychoactive substances.

## 1. Introduction

The diagnostic criteria for substance-induced psychotic disorder (SIPD) are aimed at distinguishing substance-induced psychotic states from primary psychotic disorders. According to the DSM-V, diagnosis of SIPD should be made when delusions and/or hallucinations develop in an individual during or soon after substance use or withdrawal, when the involved substance is capable of producing such symptoms, the symptoms are not better explained by a psychotic disorder, and the symptoms do not occur exclusively during the course of delirium, and when the symptoms cause significant distress or functional impairment [1]. The development of SIPD may be associated predominantly with drugs possessing strong psychotomimetic properties such as cannabis, cocaine, and methamphetamine [2]. Nevertheless, incidences of SIPD have been reported in all major classes of substances, including “downers” such as alcohol and opiates [3].

Previous studies have observed associations between development of SIPD and negative life events, such as lifetime imprisonment [4] and rehospitalisation [5]. However, the issue of particular concern is the elevated rate of transition from SIPD to permanent psychiatric conditions, namely, schizophrenia spectrum [6] and affective disorders [7]. Nationwide studies estimate that 22 to 46% of individuals diagnosed with SIPD transition to such permanent psychiatric diagnoses [6, 7]. Provided that individuals prone to SIPD may be identified by their premorbid characteristics (e.g., personality traits and sociodemographic factors) from individuals with substance use disorders (SUD), interventions could potentially target those individuals in particular. A number of studies have compared SIPD individuals with primary psychotic patients [2], but there is little data on what differentiates SUD individuals with and without SIPD. To date, only few studies have been conducted and they suggest that psychopathology may play an important role in the development of SIPD [8–11].

Unlike most substances, there has been a greater effort to document SIPD risk factors in methamphetamine users [12]. Evidence suggests that methamphetamine users who develop SIPD exhibit significantly more premorbid schizoid and schizotypal personality traits, have higher rates of depression and alcohol dependence, and report earlier age of onset of drug use than those who do not [8, 13]. Similarly, the emergence of psychotic-like symptoms in cannabis users has been linked to anxiety, negative affect (i.e., hopelessness and depression), and schizotypal personality traits [14, 15]. However, these studies have recruited occasional/recreational consumers of cannabis, not individuals presenting cannabis use disorder. Among cocaine-dependent patients, “neuroticism-anxiety” personality traits, presence of antisocial personality disorder, and early onset of consumption were associated with a higher risk of experiencing cocaine-induced psychotic symptoms [9, 11, 16, 17]. A potential caveat of studies in cocaine users is the importance given to the paranoid elements of psychosis [17]. It is noteworthy that the majority of the studies conducted in cannabis and cocaine users have investigated self-reported symptoms of psychosis and not SIPD. The fact of the matter is that SIPD is not frequent in the general population [7], which is probably why SIPD remains seldom studied.

In the present study, we retrospectively compared psychopathology, sociodemographic variables, and substance use characteristics between SIPD and non-SIPD patients that presented to a rehabilitation centre. Moreover, we set out to document the prevalence of SIPD in this population. We adopted a naturalistic approach by including polysubstance users, as they are pertinent to the clinical picture of SIPD given the high frequency of polysubstance abuse/dependence in SUDs [18].

## 2. Methods

### 2.1. Participants

A retrospective chart review was conducted at the* Clinique Nouveau Départ*, a rehabilitation centre specialised in substance abuse/dependence and comorbid psychopathologies. Data were collected from the medical records of patients (*n *= 379) newly admitted for treatment between August 2007 and October 2012. The medical director approved the screening of medical files for epidemiological purposes, and all data acquired were made anonymous. Patients were at least 18 years of age at the time of admission for treatment. Each patient was assessed for substance abuse/dependence, based on DSM-IV criteria. Substance-induced psychotic disorders were also assessed based on DSM-IV criteria. Patients with comorbid schizophrenia, bipolar disorder, and posttraumatic disorder were excluded. The clinical evaluations were performed by a physician (JPC) with expertise in addiction medicine. The study was approved by the local ethics committee.

### 2.2. Clinical Instruments

Psychometric evaluations were performed one to two weeks after admission, following the stabilization of acute withdrawal symptoms, and included standardized tests assessing consumption patterns of psychoactive substance use, such as the* Alcohol Use Disorder Identification Screening Test* (AUDIT) [19], the* Drug Abuse Screening Test* (DAST) [20], and the* Michigan Alcohol Screening Test* (MAST) [21]. Subclinical psychotic symptoms were evaluated using the psychoticism scale of the* Symptom Checklist-90R* (SCL-90R) [22]. Within the SCL-90R psychoticism scale, we further evaluated two symptoms' dimension (“schizophrenia nuclear symptoms” and “schizotypal signs”) constructed and validated by Rössler et al. [23]. Of particular interest, the “schizophrenia nuclear symptoms” dimension was obtained by adding the following items of the SCL-90R: {7} someone else can control your thoughts; {16} hearing voices other people do not hear; {35} others being aware of your thoughts; and {62} having thoughts that are not your own [23]. Depressive and anxiety symptoms were assessed using the* Beck Depression Inventory* (BDI) [24] and the* Beck Anxiety Inventory* (BAI) Beck [25]. Finally, personality traits were screened with the Millon Clinical Multiaxial Inventory-III (MCMI-III) [26]. MCMI-III personality clusters were obtained by adding the scores on the scales as follows: Cluster A (paranoid, schizoid, and schizotypal); Cluster B (antisocial, borderline, histrionic, and narcissistic); Cluster C (avoidant, dependent, and obsessive-compulsive).

### 2.3. Statistical Analyses

For continuous data (mood, personality traits, psychotic symptoms, etc.), differences between SUD individuals with and without SIPD were analyzed using analyses of variances. For dichotomous data (SUD diagnoses, gender), potential between-group differences were examined using Pearson's chi-square analyses. In non-SIPD individuals, we performed multiple hierarchical linear regression analyses to investigate associations between psychotic symptoms and clinical variables. Multiple hierarchical linear regression analyses generate models in which one or several significant predictors are hierarchically sorted according to the amount of variance (e.g., variability) they account for a given dependent variable. Psychotic symptoms were the dependent variables, and the predictors (regressors) were sociodemographic, SUD, and psychopathology variables. All analyses were performed using the* Statistical Software for Social Sciences* (version 18). Statistical significance was set at *p* < 0.05 and trends were considered at *p* < 0.1.

## 3. Results

### 3.1. Population Description

Of the 379 patients included in the study, 5% of the patients were diagnosed with SIPD (*n* = 19) and 95% of the patients were diagnosed with SUDs without SIPD (*n* = 360) (see Table 1). Compared to SUD patients without SIPD, more SIPD patients reported using cannabis and psychostimulants (including cocaine, amphetamine, and methamphetamine). However, fewer SIPD patients reported using alcohol. No differences were observed in opioid use between the two groups (see Table 2).

### 3.2. Sociodemographic Variables

Analysis of sociodemographic variables revealed that SIPD patients were significantly younger and had an earlier onset of SUD, and a higher proportion reported previous attempts to control SUD in detoxifications centers than SUDs patients without SIPD. No differences were observed in gender, education, and smoking habits between the two groups (see Table 1).

### 3.3. Clinical Characteristics and Personality Traits

SIPD patients reported feeling more distress from psychotic-like experiences, scoring higher than SUD patients without SIPD on the “schizophrenia nuclear symptoms” dimension. The “schizotypal signs” dimension, as well as the SCL-90R psychoticism scale, failed to show a difference between the two groups. SIPD patients had higher scores on the DAST questionnaire, indicating a more severe abuse of drugs and had higher MCMI Cluster B scores than SUD patients without SIPD. Moreover, SIPD patients trended towards higher MCMI Cluster A scores than SUD patients without SIPD. No differences between the two groups were observed in total MCMI Cluster C scores, as well as AUDIT, MAST, BAI, and BDI scores (see Table 3).

### 3.4. Regression Model

As shown in Table 4, in individuals without SIPD, psychoticism scores were predicted by a model including depression, anxiety, Cluster A personality traits, and cannabis abuse, which explained 45.5% of the variance. Nuclear symptoms were predicted by a model including anxiety and Cluster A personality traits, which explained 26.6% of the variance. Lastly, schizotypal signs were predicted by a model including depression, anxiety, and Clusters A and B personality traits, which explained 42.6% of the variance.

## 4. Discussion

In the present study, we compared psychopathology, sociodemographic variables, and substance use characteristics between SIPD patients and SUD patients without SIPD. We found that, compared to SUD patients without SIPD, more SIPD patients reported using cannabis and psychostimulants. By contrast, fewer SIPD patients reported using alcohol and there was no difference in opioid use between the two groups. These results are not surprising as cannabis and psychostimulants are known to have the most potential for eliciting psychotic symptoms and they are associated with the highest risk of converting to schizophrenia spectrum disorder, effects that are only minimally related or unrelated to other substances [6, 27]. Previous studies in SUD patients with cocaine as their drug of choice also reported higher rates of cannabis dependence among individuals with cocaine-induced psychosis. Similarly, there is evidence that early cannabis use is associated with severity of cocaine-induced psychosis among cocaine smokers [28]. Furthermore, two previous studies reported no differences in alcohol or opioid dependence between SIPD patients and SUD patients without SIPD [11, 29]. However, other studies examining cocaine-induced psychosis found that alcohol dependence was significantly elevated in SIPD patients, compared to individuals without SIPD [4]. The differences between studies in terms of alcohol dependence among patients with and without SIPD likely reflect baseline variations between cohorts. Finally, cannabis abuse was associated with nonspecific psychotic symptoms in non-SIPD individuals, a result consistent with literature showing that occasional cannabis smoking increases the risk of psychotic experiences [30].

We found that SIPD patients exhibited significantly greater SCL-90R schizophrenia nuclear symptoms, whereas there were no significant differences between the two groups in SCL-90R psychoticism and schizotypal signs. One major limitation of the studies investigating SIPD is that most measure psychotic symptoms without documenting SIPD diagnostics. However, we do not know whether there is a relation between self-reported psychotic symptoms and SIPD diagnosis. These results suggest that specific psychotic symptoms (i.e., schizophrenia nuclear symptoms) are in fact associated with SIPD, whereas other symptoms are not. These “schizophrenia nuclear symptoms” are items that reference Schneiderian First-Rank Symptoms [31] such as passivity experiences ({7} someone else can control your thoughts), auditory hallucinations ({16} hearing voices other people do not hear), thought broadcasting ({35} others being aware of your thoughts), and thought insertion ({62} having thoughts that are not your own). Although Schneiderian First-Rank Symptoms overall are criticized for their lack of specificity in the diagnosis of psychotic disorders, certain first-rank symptoms have been associated specifically with transition to psychosis (passivity experience) [32] and schizophrenia severity (auditory hallucinations) [33]. Together with our findings, these studies indicate that when investigating psychotic experiences, particular attention should be paid to the type of psychotic symptoms reported. We find that, within the SCL-90R psychoticism subscale, the “schizophrenia nuclear symptoms” scale should receive particular attention when assessing SIPD. In non-SIPD individuals, we found that the main predictors of self-reported psychotic symptoms were self-reported anxiety-depressive symptoms. This suggests that self-reported psychotic symptoms may be conceptualized in a continuum with anxiety, where psychotic manifestations are not necessarily associated with a risk of transitioning to psychotic disorders. Considering the emphasis placed on identifying individuals at risk of developing schizophrenia, as well as the volume of ongoing research investigating the relationship between cannabis use and self-reported psychotic experiences, our results indicate that more attention should be paid to qualitative distinctions between diverse psychotic manifestations reported in the general population, and in particular in SUDs populations.

Although not a statistically significant difference, we found a trend towards elevated MCMI Cluster A personality traits in SIPD individuals, a result generally consistent with previous studies. For example, one study found that methamphetamine users who developed psychosis exhibited significantly more premorbid schizoid and schizotypal personality traits than those who did not [8]. By contrast, Roncero et al. [16] did not find a difference in paranoid, schizoid, or schizotypal personality disorders between individuals with and without cocaine-induced psychosis. We also observed a significant association between Cluster A personality traits and self-reported psychotic symptoms in non-SIPD individuals, results that were confirmed in cannabis users without SIPD, where high scoring schizotypes were found to be more likely to experience psychosis-like phenomena [14, 34].

We found that SIPD patients exhibited significantly more MCMI Cluster B personality traits, relative to SUD patients without SIPD. Consistent with our data, Roncero et al. [11] reported that, among cocaine-dependent patients, the presence of antisocial personality disorder was associated with the risk of experiencing SIPD. Tang et al. [29] also found that individuals with cocaine-induced psychosis presented with significantly higher rates of antisocial personality disorder, compared with cocaine-dependent subjects without SIPD. In the case of the potential association between borderline personality disorder and SIPD (e.g., cocaine), the evidence has been mixed thus far [9, 11, 16]. It is noteworthy that, in the study from Roncero et al. [16], Cluster C personality traits were not related to SIPD, which is also consistent with our results.

We did not find significant differences in anxiety or depressive symptoms among SIPD patients, relative to SUD patients without SIPD. Results reported in the literature have been mixed. For instance, Roncero et al. [9] found that the personality trait “neuroticism-anxiety” was associated with SIPD among cocaine-dependent patients. Moreover, Chen et al. [8] reported that individuals with methamphetamine-induced psychosis exhibited significantly more major depression than those without SIPD. On the other hand, Tang et al. [29] and Roncero et al. [4] did not find a significant difference in major depression between cocaine-dependent patients with and without SIPD. While we found no association between anxiodepressive symptoms and SIPD in the current study, we did find an association between anxiodepressive symptoms and self-reported psychotic symptoms in SUD individuals without SIPD. We may therefore hypothesize that anxiety and depression are associated with unspecific self-reported psychotic symptoms, but not with SIPD more specifically.

Analysis of sociodemographic and SUD variables showed that SIPD patients were significantly younger at the time of sampling and they had an earlier age of onset of SUD. Furthermore, SIPD patients reported significantly more past detoxification attempts and higher drug (but not alcohol) use severity. These results are consistent with previous studies. Roncero et al. [4] found that patients with cocaine-induced psychosis had a significantly lower age of onset of SUD and longer duration of dependence, relative to individuals without SIPD and those with transient psychosis. In addition, cocaine-induced psychosis patients trended towards being younger and using higher quantities of substances, relative to the other groups. Likewise, there is evidence that younger age at onset of alcohol dependence was associated with an increased risk of developing alcohol-induced psychosis [35].

In spite of a fair sample of SUD individuals (*n* = 379), the present study is limited due to the low number of SIPD patients in the cohort (5%), which is a potential reason why we did not observe certain significant between-group effects, such as the relationship between SIPD and Cluster A personality traits. However, this is a real-world figure since previous research on cocaine-induced psychosis revealed a prevalence of 5% to 40% of SIPD among cocaine users [36, 37]. As for alcohol-induced psychosis, the lifetime prevalence is estimated to be much lower (0.5% [35]). The cross-sectional design used in this study is another limitation, as we could not determine whether symptoms were truly premorbid (direction of relationship) and therefore we were not able to examine the influence of the duration of psychotic symptoms. Finally, the factor structure of the SCL-90R [38, 39], and especially the “psychoticism” dimension, has often been criticized [40]. Nonetheless, the “schizophrenia nuclear symptoms” and “schizotypal signs” subscales [23], which allow distinguishing between types of psychotic symptoms, have been successfully employed to investigate subclinical psychotic symptoms in the general population in recent studies [41–44].

## 5. Conclusion

In the present study, we found that more SIPD patients reported using cannabis and psychostimulants than SUD patients without SIPD. SIPD patients also reported more personality disorders than SUD patients without SIPD. More importantly, we found that SIPD patients reported higher scores on the “schizophrenia nuclear symptoms” dimension than SUD patients without SIPD. These results highlight the importance for future studies to consider the specific types of psychotic symptoms reported when assessing the psychotomimetic effects of psychoactive substances.

## Figures and Tables

**Table 1:** Socio-demographic characteristics of the study population.

	Total study population	SUDs without SIPD	SIPD	Significance
Sample size	379	360	19	
Sex, % male	72.6	72.2	78.9	*p* > 0.1
Age, mean (SE)	41.1 (0.72)	**41.5 (0.74)**	**33.3 (2.50)**	*t*(377) = 2.51, *p* = 0.013
Education, years (SE)	13.9 (0.10)	13.93 (0.11)	13.21 (0.42)	*p* > 0.1
Age of Onset of SUD, years (SE)	25.62 (0.63)	**25.94 (0.11)**	**19.68 (1.61)**	*t*(372) = 2.15, *p* = 0.033
Smoking when admitted, %	66.8	66.1	78.9	*p* > 0.1
Past detox reported (>1), %	38.0	**36.4**	**63.2**	*x* ^2^ = 5.50, *p* = 0.019

SE = standard error of the mean; SUD = substance use disorder, SIPD = substance-induced psychotic disorder.

**Table 2:** Prevalence of SIPD diagnosis by substance (including poly use).

Substance Use	SUDs without SIPD		SIPD		Chi-Square		
	*N*	%	*N*	%	Value	df	*p*
Alcohol							
No	110	30.56	11	57.89	6.207	1	**0.013**
Yes	250	69.44	8	42.11			
Cannabis							
No	268	74.44	7	36.84	12.82	1	**0.000**
Yes	92	25.56	12	63.16			
Opioids							
No	296	82.22	18	94.74	1.989	1	>0.1
Yes	64	17.78	1	5.26			
Psychostimulants							
No	264	73.33	7	36.84	11.79	1	**0.001**
Yes	96	26.67	12	63.16			

Opioids = heroin, prescription pain killers; Psychostimulants = cocaine, amphetamines, methamphetamines; SUD = substance use disorder; SIPD = substance-induced psychotic disorder.

**Table 3:** Clinical characteristics of SUD individuals with SIPD.

	SUDs without SIPD		SIPD	
	(*n* = 360)		(*n* = 19)	
	Mean	(SE)	Mean	(SE)
SCL-90R Psychoticism	63.80	(0.62)	65.63	(3.17)
Schizophrenia Nuclear Symptoms	**1.65**	**(0.14)**	**3.06**	(0.81)^*∗*^
Schizotypal Signs	7.04	(0.43)	9.17	(1.78)
MCMI Cluster A	**52.45**	**(0.97)**	**59.93**	(2.95)^*#*^
MCMI Cluster B	**55.75**	**(0.63)**	**61.89**	(2.79)^*∗*^
MCMI Cluster C	53.56	(0.75)	52.76	(2.97)
AUDIT	17.37	(0.62)	15.94	(2.82)
MAST	18.97	(0.83)	19.22	(3.76)
BAI	18.66	(0.75)	19.21	(3.65)
BDI	20.46	(0.63)	19.79	(2.30)
DAST	**6.19**	**(0.30)**	**10.44**	(1.30)^*∗∗*^

^#^Trend at *p* < 0.1.

^*∗*^Statistically significant at *p* < 0.05.

^*∗∗*^Statistically significant at *p* < 0.01.

AUDIT = Alcohol Use Disorder Identification Test; BAI = Beck Anxiety Inventory; BDI = Beck Depression Inventory; DAST = Drug Abuse Screening Test; MAST = Michigan Alcohol Screening Test; MCMI Cluster A, B, C = personality traits/disorders; Cluster A (schizoid, paranoid, schizotypal); Cluster B (borderline, antisocial, histrionic, narcissistic); Cluster C (avoidant, dependent, compulsive); SCL-90R = Symptoms Checklist Revised; SUD = substance use disorder; SIPD = substance-induced psychotic disorder.

**Table 4:** Regression of SUD individuals without SIPD (*n* = 360).

Predictors of Psychoticism scores	*r* ^2^	*β*	Statistical significance
*SCL-90-R Psychoticism*			
Model	0.455		*F* = 75.960; *p* < 0.001
BDI		0.389	*t* = 7.948; *p* < 0.001
BAI		0.218	*t* = 5.519; *p* < 0.001
MCMI Cluster A		0.107	*t* = 3.782; *p* < 0.001
Cannabis abuse		2.483	*t* = 2.395; *p* = 0.017
*Schizophrenia Nuclear Symptoms*			
Model	0.266		*F* = 64.389; *p* < 0.001
BAI		0.082	*t* = 9.105; *p* < 0.001
MCMI Cluster A		0.022	*t* = 3.064; *p* = 0.002
*Schizotypal Signs*			
Model	0.426		*F* = 66.379; *p* < 0.001
BDI		0.202	*t* = 7.349; *p* < 0.001
BAI		0.100	*t* = 4.454; *p* < 0.001
MCMI Cluster A		0.063	*t* = 3.889; *p* < 0.001
MCMI Cluster B		0.058	*t* = 2.585; *p* = 0.010

BAI = Beck Anxiety Inventory; BDI = Beck Depression Inventory; MCMI Cluster A, B, C = personality traits/disorders; Cluster A (schizoid, paranoid, schizotypal); Cluster B (borderline, antisocial, histrionic, narcissistic); Cluster C (avoidant, dependent, compulsive).
